# Dynamics of water bound to crystalline cellulose

**DOI:** 10.1038/s41598-017-12035-w

**Published:** 2017-09-19

**Authors:** Hugh O’Neill, Sai Venkatesh Pingali, Loukas Petridis, Junhong He, Eugene Mamontov, Liang Hong, Volker Urban, Barbara Evans, Paul Langan, Jeremy C. Smith, Brian H. Davison

**Affiliations:** 1 Biology and Soft Matter Division, Oak Ridge National Laboratory, Oak Ridge, Tennessee 37831 United States; 2 Biosciences Division, Oak Ridge National Laboratory, Oak Ridge, Tennessee 37831 United States; 3 Chemical and Engineering Materials Division, Oak Ridge National Laboratory, Oak Ridge, Tennessee 37831 United States; 4 Chemical Sciences Division, Oak Ridge National Laboratory, Oak Ridge, Tennessee 37831 United States; 5 Department of Biochemistry and Cellular and Molecular Biology, University of Tennessee, Knoxville, Tennessee 37996 United States; 6 School of Physics and Astronomy & Institute of Natural Sciences, Shanghai Jiao Tong University, Shanghai, 200240 China

## Abstract

Interactions of water with cellulose are of both fundamental and technological importance. Here, we characterize the properties of water associated with cellulose using deuterium labeling, neutron scattering and molecular dynamics simulation. Quasi-elastic neutron scattering provided quantitative details about the dynamical relaxation processes that occur and was supported by structural characterization using small-angle neutron scattering and X-ray diffraction. We can unambiguously detect two populations of water associated with cellulose. The first is “non-freezing bound” water that gradually becomes mobile with increasing temperature and can be related to surface water. The second population is consistent with confined water that abruptly becomes mobile at ~260 K, and can be attributed to water that accumulates in the narrow spaces between the microfibrils. Quantitative analysis of the QENS data showed that, at 250 K, the water diffusion coefficient was 0.85 ± 0.04 × 10^−10^ m^2^sec^−1^ and increased to 1.77 ± 0.09 × 10^−10^ m^2^sec^−1^ at 265 K. MD simulations are in excellent agreement with the experiments and support the interpretation that water associated with cellulose exists in two dynamical populations. Our results provide clarity to previous work investigating the states of bound water and provide a new approach for probing water interactions with lignocellulose materials.

## Introduction

The role of water in the plant cell wall is of fundamental importance for plant science as well as many industrial applications of wood-based materials. This is because the physical and mechanical properties of lignocellulose are strongly influenced by the interactions of water with each component biopolymer within the cell wall ultrastructure^1,2^. The majority of the plant cell is comprised of water, which is essential for many of the biochemical and physiological processes in plants^3^. From the technological perspective, the water-wood relationship can affect durability and this can negatively impact dimensional stability, cause splitting, mold growth, wood decay and also corrosion of embedded metals^4^. Equally, these associations are crucial for cellulose adhesion to other materials for producing cellulose nanocomposites or reinforced thermoplastics, and also for paper production^5,6^.

Understanding how water interacts with lignocellulose is critical to understand the underlying processes that change biomass morphology during different pretreatment regimes for biofuels production^7^. Water plays roles in mass transfer of reactants and products^8^, in determining the heat transfer efficiency^9,10^, and acting to physically disrupt biomass in explosion type pretreatments. In addition, it can also act as a reactant in pretreatments by providing mildly acidic conditions at elevated temperature^11,12^. Knowledge of the dynamical properties of bound water and how it partitions in the cellulose supramolecular structure can further enhance our understanding of water’s role in these processes and potentially lead to more efficient pretreatment approaches.

Although cellulose-water interactions have major fundamental as well as technological importance, and have been studied over many decades, the behavior of water on the crystalline cellulose surfaces has not been fully characterized on the molecular level. In plant cell walls, the interaction of water with cellulose spans length scales from hydrogen bonding at the molecular scale to hydration of cellulose microfibrils and the interfibrillar hydration of the surrounding matrix components (hemicelluloses, lignin, pectins). It is well accepted that there are at least two different types of water present in lignocellulose: “free” water assigned to the capillary water within cell lumens, and “bound” water interacting with the component cell wall polymers. Differential scanning calorimetry studies further resolved two types of bound water in cellulose purified from different sources and based on these studies categorized bound water into “freezing bound” and “non-freezing bound” fractions^13^.

NMR spectroscopy has been used to investigate the states of water in lignocellulose, and isolated cell wall components. One-dimensional (1D)^1^H NMR resolves a slowly relaxing component that is characteristic of lumen water and a second component that is assigned to water in the cell walls^14–16^, and using two-dimensional (2D) *T*_2_ – *T*_1_ NMR it is possible to resolve two different states of bound water in spruce wood^17^. In addition, a solid state NMR study of water dynamics in lignocellulose revealed that pectin significantly affects water mobility in plant cell walls^18^. In other work, comparison of molecular dynamics (MD) simulations to solid state CP/MAS^13^C NMR results found that two different crystallographic planes of cellulose are non-equivalent with respect to how they interact with water^19^.

Neutron scattering techniques have also been used to study cellulose - water interactions. Inelastic neutron scattering applied to study the interaction of crystalline and amorphous celluloses with water revealed a common dynamic signature for disordered, water-accessible regions of several native celluloses^20,21^. Here, we report on quasi-elastic neutron scattering (QENS), a non-destructive technique, to study water - cellulose dynamics. QENS has been used to study relaxation and diffusive dynamics of a variety of biological systems on a pico- to nanosecond timescale. These include biomolecules such as proteins, t-RNA, and DNA, and also their hydration water^22–26^. QENS is especially useful for studying water dynamics in these biomolecules because it can resolve the spatial differences in the dynamics of the hydration water and distinguish between localized rotational motions of chemical groups and long-range translational motions^27^. This is because of the dependence of the observed relaxation times on the wave vector Q, which cannot be accessed with other spectroscopic techniques, such as NMR^28,29^.

Several studies have utilized bacterial cellulose produced by *Acetobacter xylinus* as a model material to investigate specific aspects of plant cell wall structure and chemistry^30^. Both plant and bacterial cellulose are chemically identical and exhibit a hierarchical organization. Differences in their nanoscale structures include a larger elementary fibril size (5–6 nm) for bacterial cellulose compared to plant cellulose (2–3 nm), and the crystalline isomorph is Iα (60–70%) in bacteria, while in typical plant cell walls, the predominant isomorph is Iβ. In addition, the microfibrils extruded by the bacteria form a random network, with its microfibrillar structure unobscured by other polymers. In the plant cell, matrix polysaccharides, together with spatial and temporal constraints, modulate microfibril elongation and architecture^31^. We previously reported on the mechanical and dynamical properties of bacterial cellulose hydrated in D_2_O using QENS and MD simulation. We characterized the motions in cellulose, their dependence on temperature and hydration, and how these motions affect the mechanical properties of the microfibril^32^. Unexpectedly, cellulose was found to be more rigid in the hydrated state than in the dry state, and evidence was found that in hydrated cellulose the cellulose chains are more closely packed and water molecules bridge the chains through hydrogen bonding interactions. Therefore, it is of considerable interest to investigate if the microscopic dynamical properties of the water-associated cellulose can give new insight into cellulose - water relations in the hygroscopic range.

Here, we address the nature of these interactions using deuterium labeling, neutron scattering and molecular dynamics simulation, revealing water diffusion on cellulose at the atomic scale. Perdeuterated cellulose produced from *Gluconacetobacter sp*. enabled us to directly measure the QENS signal of the H_2_O associated with the cellulose fibers because the large difference in the incoherent scattering cross-section of hydrogen and its isotope deuterium (~40 fold) makes it possible to separate dynamics of deuterated biomolecules from its hydration water^22^. The main result of our work is that we can unambiguously detect the presence of two populations of bound water associated with cellulose. The first population is non-freezing water associated with the cellulose surfaces and the second is weakly bound water in a confined state, consistent with water trapped between microfibrils. Our results are discussed in the context of the other recent studies using DSC^33,34^ and NMR^17^ to address an on-going controversy about the state of bound water in crystalline cellulose.

## Materials and Methods

### Sample preparation

The procedure for growth of *Acetobacter xylinus* sub sp. *sucrofermentans* (ATCC 700178) in D_2_O-based media and purification of deuterated cellulose has been described previously^35,36^. The final growth medium contained ~97.6% D_2_O media with *D*_8_-glycerol as the carbon source. Briefly, the crystalline cellulose pellicles secreted by the bacteria were purified by washing in 1% NaOH to remove bacterial debris, followed by washing with H_2_O until the pH of the surrounding solvent reached ~pH 7. The purified cellulose was then ground to a slurry and lyophilized to a constant weight to ensure that all water was removed.

### Small-angle neutron scattering (SANS)

SANS measurements were performed with the CG-3 Bio-SANS instrument at the High Flux Isotope Reactor (HFIR) facility of Oak Ridge National Laboratory^37^. Sample-to-detector distances of 2529 mm, 6829 mm and 15329 mm were employed to cover the Q range 0.003 < *Q* (Å^−1^) < 0.4, at 6 Å neutron wavelength. The scattering vector, $Q=(4\pi /\lambda )sin\theta$, describes the relation of *Q* to *λ*, neutron wavelength and 2*θ*, the scattering angle. The center of the area detector (1 m × 1 m GE-Reuter Stokes Tube Detector) was offset by 350 mm from the beam. The relative wavelength spread *Δλ*/*λ* was set to 0.15. The scattering intensity profiles *I*(*Q)* versus *Q*, were obtained by azimuthally averaging the processed 2D images, which were normalized to incident beam monitor counts, and corrected for detector dark current, pixel sensitivity and scattering from the quartz cell.

The cellulose was hydrated under isopiestic conditions with 40:60 v:v D_2_O:H_2_O, the D_2_O concentration that was previously determined to be the scattering match point for protiated bacterial cellulose^35^. Prior to data fitting, a flat background was subtracted from the scattering curves to remove the incoherent scattering contribution. A power-law was fit to the dry sample, whereas the hydrated bacterial cellulose sample was best modeled as the convolution of a power-law and a correlation peak. The power-law intensity relation is $I(Q)={(P\cdot Q)}^{-\alpha }+B$ where *P* is the power-law scaling factor, α is the power-law exponent and *B* is the flat background. The correlation peak was fit to a Lorentzian peak form:

1 $$I(Q)=A\times (1/\pi )\times \frac{(w/2)}{({(w/2)}^{2}+{(Q-\sigma )}^{2})}$$

where *A* is the scale factor, *w* is the peak width and *σ* is the peak center.

### Wide-Angle X-ray Diffraction (WAXD)

WAXD measurements were performed using a theta-theta goniometer Bruker D5005 instrument using Cu *K*α radiation (*λ* = 1.542 Å) operating at 40 keV and 40 mA. The sample-holder was a flat glass-slide with a circular trough in the center for the sample. The angular resolution continuously increases from low to high 2*θ* angles (5° to 90°) as a result of using fixed-width slits for collimation. The angular resolution was defined using a 1 mm slit on the incident arm with two slits sandwiching a *Ni* absorber to remove Cu *K*β X-rays. The resolution on the analyzer arm was defined with a 1 mm slit closer to sample and a 0.6 mm slit just prior to the detector. The width of the diffraction peaks associated with specific reflecting planes (*hkl*) having a repeat spacing of *d*_*hkl*_ were used to estimate the crystallite size (or dimension), *L*_*hkl*_ using the Scherrer equation^38,39^:

2 $${L}_{hkl}=\,\frac{0.9\lambda }{{\beta }_{1/2}\,\cos \,{\rm{\theta }}}$$

where *λ* is the X-ray wavelength in Å, *β*_1/2_ is the angular full-width at half maximum (FWHM) intensity in radians (*hkl*) of the line profile; and *θ* is the scattering angle. The spacing *d*_*hkl*_ of these planes (*hkl*) is obtained by Bragg’s law^38,39^:

3 $${d}_{hkl}=\frac{\lambda }{2\,\sin \,{\rm{\theta }}}$$

Of the many diffraction peaks observed, three main characteristic peaks of the cellulose I microfibril cross-sectional correlations^40–42^ were present and one peak from along the fibril axis. These peaks were fit using pseudo-Voigt functions (100) around 14.8°, (010) at ~16.9° and (110) around ~22.9° for cross-sectional microfibril correlations and ($11\bar{2}$) at ~20.7° for along the fibril axis.

### Quasi-elastic Neutron Scattering (QENS)

QENS measurements were performed using BASIS, the Backscattering Spectrometer, at the ORNL Spallation Neutron Source (SNS) in an energy range of +/−100 µeV (~6 ps) with a resolution of 3.5 µeV, full-width at half maximum (FWHM), (~400 ps) and a Q range of 0.2–2.0 Å^−1^ ^43^. The lyophilized cellulose was hydrated by vapor exchange under isopiestic conditions to 0.22 g of H_2_O/g cellulose and sealed in annular aluminum cans. QENS measurements were performed from low temperature to high temperature to avoid hysteresis effects that could occur if the measurement were performed in the opposite direction. QENS data analysis was carried out using the DAVE software package^44^.

For quantitative analysis of the spectral broadening in the QENS spectra as a function of Q, the spectra at each Q value were fitted using the following expression as a function of energy transfer, E:

4 $$I(Q,E)=[x(Q)\delta (Q,E)+(1-x(Q))S(Q,E)]\otimes R(Q,E)+[{C}_{1}(Q)E+{C}_{2}(Q)]$$

where *x* is the fraction of the elastic scattering in the spectrum, δ(Q, E) is a delta-function describing the elastic peak at zero energy transfer, S(Q, E) is a model scattering function, R(Q, E) is the resolution function (derived from the data measured at low temperature), C_1_ and C_2_ are Q-dependent constants in energy transfer in the last two terms in the bracket representing a linear background. Eq. (4) is rather general, and the dynamic features of the system are specified by the model scattering function, which often can include either one or two components; in the latter case with spectral weight of *p* and (1−*p*) for the narrow and broad component, respectively:

5 $$S(Q,E)=p(Q){S}_{1}(Q,E)+(1-p(Q)){S}_{2}(Q,E)$$

At a given Q, the simplest functional form for S_1_(Q, E) and S_2_(Q, E) in the energy space is a Lorentzian, corresponding to a Fourier-transformed exponential decay in the time space describing a simple diffusion process. However, in many complex systems, diffusion is better described by a “stretched” exponential, leading to “stretched” non-Lorentzian signal shape in the energy space. The amount of information in typical QENS spectra is usually insufficient for data fits with more than one “stretched” component. The practical choice between a single “stretched” component fit model and two Lorentzian components fit model can be guided by converting the S(Q, E) data into the dynamic susceptibility, which here was obtained from the normalized QENS data in the energy transfer range from 0 to 100 μeV according to the following equation:

6 $$\chi ^{\prime\prime} (Q,E)\propto S(Q,E)/{n}_{B}(T)$$

where *n*_*B*_ *(T)* is the temperature Bose Factor. QENS data are presented as the imaginary part of the susceptibility (χ″(Q, E) versus E (μeV)).

A jump diffusion model^45^ was used to estimate the diffusion coefficient of water associated with cellulose. Using this, the Q^2^ dependence of the Equation 7 is

7 $${{\rm{\Gamma }}}_{\frac{1}{2}}({\rm{Q}})=\frac{\hslash }{{\tau }_{0}}[1-\frac{1}{1+D{{\rm{Q}}}^{2}{\tau }_{0}}]$$

where Γ_1/2_ (Q) is the Q dependent half-width at half maximum (HWHM) of the S(Q,E) components, *D* is the diffusion constant, ${\tau }_{0}$ is the residence time between successive jumps, and *ħ* is the reduced Plank’s constant. The jump length, *l*, of molecules is related to *D* and τ_0_ as,$\,l=\sqrt{(6D{\tau }_{0})}$.

### Molecular Dynamics (MD) Simulations

The simulation trajectories first reported in ref. ^32^ were analyzed. The model contains four aligned cellulose microfibrils. Each microfibril contains 36 chains with a degree of polymerization of 80. The system was solvated at 0.20 g water/g cellulose to match the experiments. Since cellulose Iα^46^ is the predominant allomorph of bacterial cellulose, this allomorph was modeled here. The simulations were performed employing the GROMACS^47^ software and the CHARMM C36 carbohydrate force field^48,49^. The TIP4P^50^ water model was chosen because its self-diffusion coefficient is in good agreement with experiments. Bonds involving hydrogen atoms (C-H and O-H) were constrained to their equilibrium values and a 2 fs time step was used. Periodic boundary conditions were employed together with the Particle Mesh Ewald (PME) algorithm^51,52^ and a cutoff of 12 Å for the neighbor searching and real-space electrostatics. A switch function between 9–10 Å was used for the van der Waals interactions. The V-rescale thermostat^53^ (τ = 0.1 fs) and Parrinello-Rahman barostat^54^ (τ = 1 fs) were employed. Each system was simulated for a total of 11 ns at three temperatures: 213 K, 243 K and 263 K and at atmospheric pressure. The data from the final 5 ns of each simulation were analyzed, once the system had reached equilibration.

## Results and Discussion

### Cellulose structure and morphology

The structural properties of cellulose produced by *Gluconacetobacter sp*.^35,36^ were characterized as a prerequisite to the investigation of the dynamic properties of cellulose – water interactions. SANS was used to compare the nanostructure and bulk morphology of dry and hydrated cellulose. The cellulose was hydrated with 40:60 v:v D_2_O:H_2_O, the contrast match point for protiated bacterial cellulose^35^, to selectively highlight the effect of hydration on its structure.

**Figure 1:**
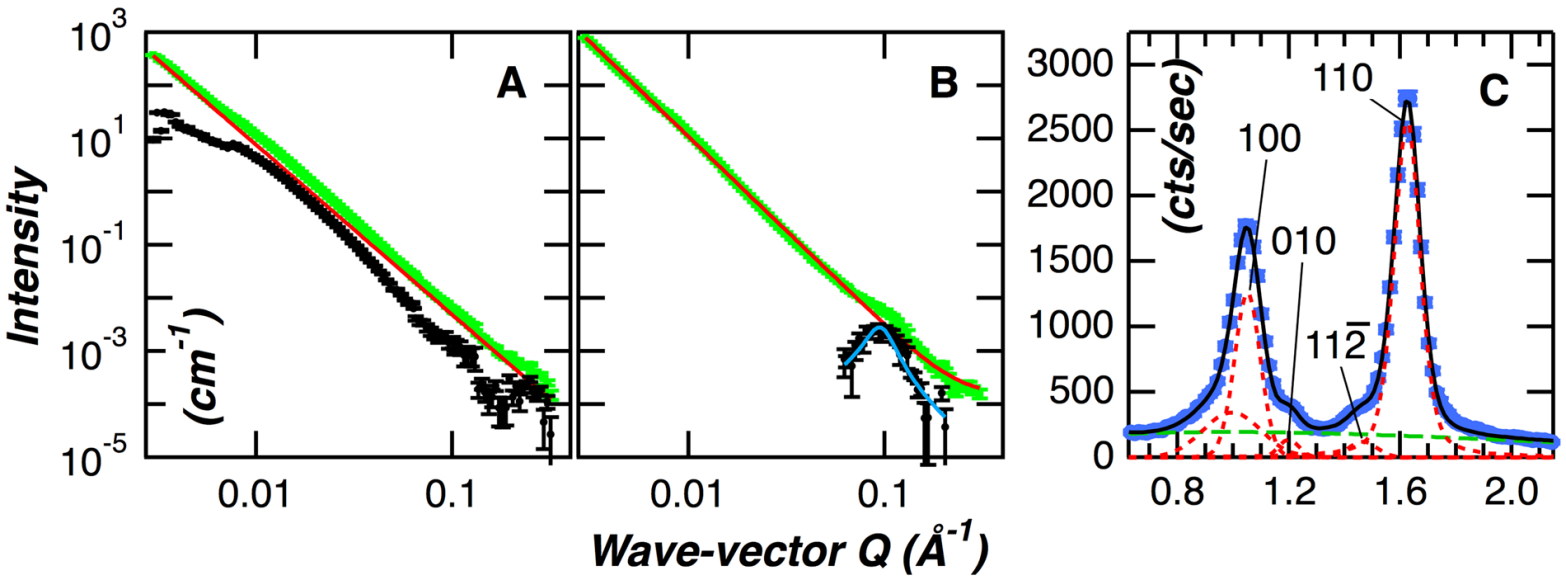
Analysis of the structure and morphology of dry and hydrated bacterial cellulose. *Panel A*. SANS profile of dry bacterial cellulose. The experimental SANS data is shown as green square symbols, the power-law fit is shown as a solid red line, deviation from the power-law behavior, evident after subtraction of the underlying power-law is shown as black filled circles. *Panel B*. SANS profiles of hydrated bacterial cellulose. The color scheme of the curves is the same as panel A. *Panel C*. Powder X-ray diffraction pattern of bacterial cellulose hydrated in H_2_O. Experimental data is shown as blue square symbols, green dashed line is the background, and the red dashed lines show the peak positions obtained from data fitting as described in the Materials and Methods section.

In both the dry and hydrated cellulose, the scattering profiles monotonically increase with decreasing *Q* over the *Q* range measured (Fig. 1A,B). The *Q* exponent of the power-law profile increased from 3.20 ± 0.01 to 3.55 ± 0.01 after hydration of the sample, indicating that the cellulose surface transitioned from a rough to smoother morphology after the addition of water. Deviation from the power-law behavior is evident in both the curves, and is more clearly observed after subtraction of the underlying power-law from each scattering curve (black dots in Fig. 1A,B). In the dry sample, the excess scattering after subtraction of the power-law appears in the mid-Q region and likely represents the mesoscale organization of the bacterial cellulose^35^. The SANS curve for the hydrated sample displayed power-law behavior over the majority of the measured Q-range and only deviated in the high Q region (Q >~0.08 Å^−1^). After subtraction of the underlying power-law only a correlation peak remained (Fig. 1B). This feature, centered at *Q* = 0.094 ± 0.001 Å^−1^, represents a characteristic repeat distance in the material, and is equivalent to a real-space repeat distance of 67.0 ± 0.6 Å. Here, we interpret the repeat distance as the center-to-center distance between adjacent cellulose microfibrils. The X-ray diffraction (XRD) pattern shown in Fig. 1C, obtained for the dried material, identified three major peaks at 14.7°, ~16.9° and ~22.9°. These peak positions are similar to those previously reported for cellulose Iα and can be associated with the 100, 010 and 110 crystalline planes of the cellulose microfibril^36^. The difference in the relative intensities of the 100 and 010 reflections indicates a preferred crystalline orientation in the samples rather than a random orientation of crystallites^55^. A previous study reported a similar result, although the relative intensities of the 100 and 010 reflections were reversed compared to this study, and found that the preferential orientation of the cellulose crystals was related to the dehydration process^56^. The crystalline width of the cellulose microfibril calculated from the 110 plane was 50 ± 1 Å. The combined SANS and XRD analyses indicate that the space between two neighboring microfibrils is 17 Å, calculated from a center-to-center distance of 67 Å for adjacent microfibrils from SANS minus a microfibril width of 50 Å from XRD. In summary, we can describe bacterial cellulose as a mesoporous material with ~2 nm interfibrillar spaces or pores that transitions from a relatively rough surface in the dry state to a smooth surface after hydration.

### Water states in crystalline cellulose

We used QENS to probe the dynamics of water associated with cellulose. The cellulose used for these measurements was grown in a deuterated form by *Gluconacetobacter sp*.^35,36^. By deuterium labeling the aliphatic hydrogens of the glucose residues their contribution to the scattering signal was greatly reduced, thus highlighting the scattering contribution of the water. The deuterated cellulose sample was hydrated with H_2_O using the vapor exchange method to ensure a uniform distribution of H_2_O on the cellulose microfibrils. At a hydration level of 22% used here, the amount of water associated with cellulose is below the fiber saturation point (FSP) (32%)^3,57^, indicating all water present is in the bound state. Additionally, the amount of bound water is above the percolation threshold (~18%), and the water is therefore able to freely diffuse on the cellulose surface^58^.

**Figure 2:**
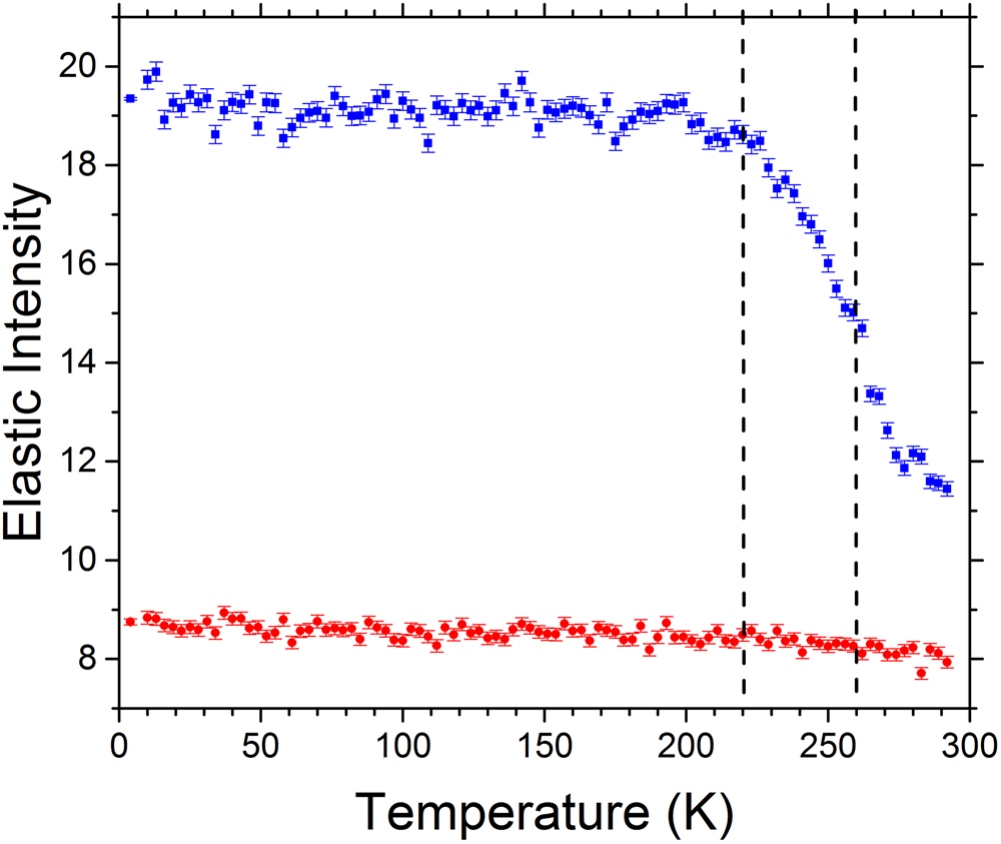
Elastic intensity scans of dry and hydrated deuterated cellulose. Data collected at 0.9 Å^−1^ are shown. The scans at other Q values showed a similar trend. The data curves with blue squares and red circles represent the hydrated and dry samples, respectively. The dashed lines denote inflection points in the curves at 220 and 260 K in the hydrated cellulose sample.

Temperature dependent elastic intensity scans provide a quantitative measure to determine the onset of transitions in the dynamics, *i.e*. at what temperatures the dynamics of different water populations enter the time window of the spectrometer (determined by its energy resolution), leading to the decrease of the elastic intensity (Fig. 2). Scans recorded in the Q-range from 0.5 to 1.3 Å^−1^ remained essentially unchanged for the dry sample over the temperature range measured (4–292 K), indicating the absence of activated processes, except for thermal vibrations. In the case of the hydrated sample, the initial intensity of the elastic peak was higher due to scattering contribution from the H_2_O and showed a weak linear decrease with temperature up to T ~210–220 K. Above this temperature, a sharp decrease in the elastic intensity is observed which is indicative of the onset of anharmonic dynamics, followed by a sharp intensity drop at ~260 K, consistent with the activation of a further relaxation process. We can infer that the temperature dependent changes in the elastic signal observed in the hydrated sample were dominated by hydration water due to the absence of any similar trend in dry deuterated cellulose.

The dynamics of bound water has been studied in a wide range of materials including biomolecules, polymers, and inorganic systems. In the case of biomolecules such as hydrated protein powders and polysaccharide gels (at less than 40% hydration), a single transition in the melting temperature of bound water is observed, indicating that water behaves as a uniform population^59,60^. This is clearly different to the data presented here. On the other hand, studies of water dynamics associated with mesoporous materials such as MCM41 and SBA15 silica show a similar trend to data obtained in this study. The behavior of water associated with these materials has been studied using a variety of techniques including NMR, DSC, and QENS and reveal that bound water can be separated into a component that tightly interacts with the silica surfaces and a second component of more loosely bound water that accumulates in the pores of the material^61–64^. These materials are characterized as having large hydrophilic surfaces with well-defined pores in the range of 1–10 nm, that can be controlled by the synthesis conditions^65,66^. In addition, there is a direct correlation between pore size and freezing point suppression, smaller pores having a greater suppression of the freezing point of confined water. For instance, freezing points of 252 K and 237 K were obtained in silica with the average pore size of 10 nm and 3 nm, respectively^67^.

In the present study, the temperature dependent elastic intensity scan data (Fig. 2) clearly show two transitions arising from the water component of the hydrated cellulose and the structural properties of the material are consistent with a mesoporous material with ~2 nm interfibrillar pores. Therefore, it reasonable to propose that there are in fact two types of water that are associated with cellulose. Using the nomenclature first proposed by Nakamura *et al*.^13^, the first type of water is “non-freezing bound” water that gradually becomes mobile with increasing temperature (appreciably above 220 K) and can be related to bound surface water. The second population is less-tightly bound compared to the surface water and is consistent with water confined in pores that abruptly becomes mobile at ~260 K. This population of water is “freezing bound” water that accumulates in the narrow spaces between the microfibrils.

### Dynamics of bound water

**Figure 3:**
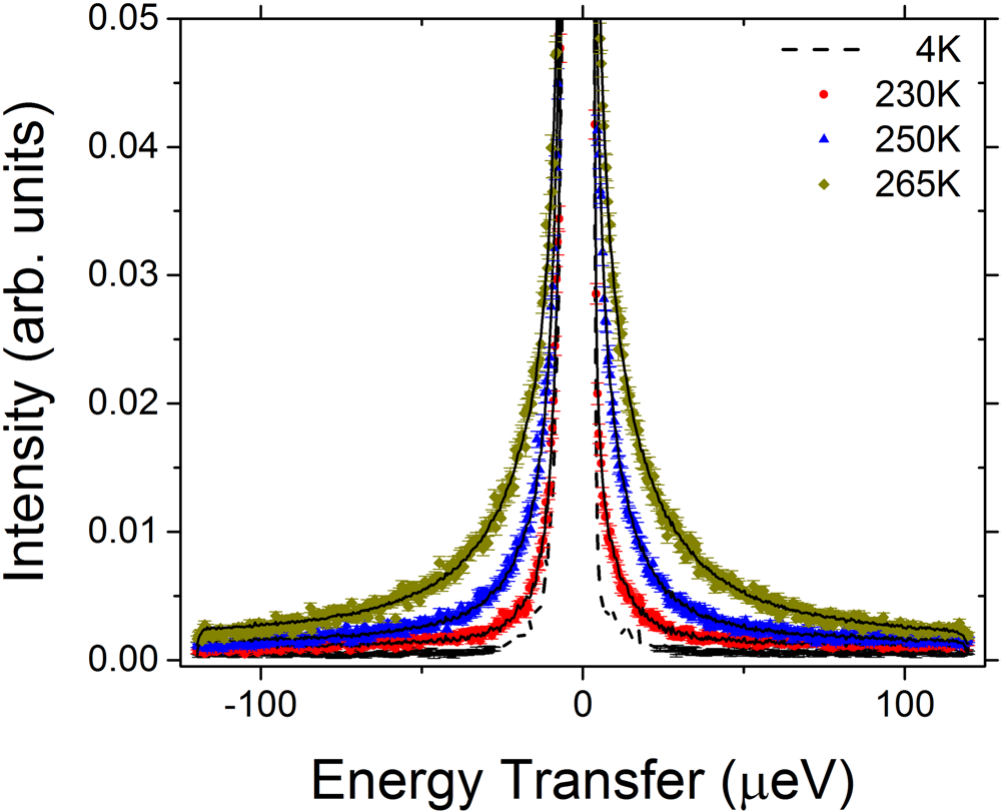
QENS spectra of hydrated cellulose at different temperatures. QENS spectra are shown for Q = 0.9 A^−1^, other Q values show a similar trend. The closed shapes (see legend) are the measured neutron intensity as a function of the energy transfer *E* at 230, 250 and 265 K. The solid black lines are the fitted curves using a two-Lorentzian model, as described in the text. The dashed black line is the resolution function.

In order to better understand the processes that give rise to the inflections in the slopes revealed by the elastic intensity scans for the hydrated deuterated cellulose, three temperature points, 230, 250 and 265 K, were chosen for a more detailed study of the dynamics of the system. The measurements at 230 K and 250 K represent temperatures between the first and second transitions in the slope observed in the elastic scan data (Fig. 2). The measurement at 265 K represents a temperature above the second transition. Representative QENS spectra at Q = 0.9 Å^−1^ are shown in Fig. 3. The elastic intensity peak is centered near zero energy transfer (0 μeV).

**Figure 4:**
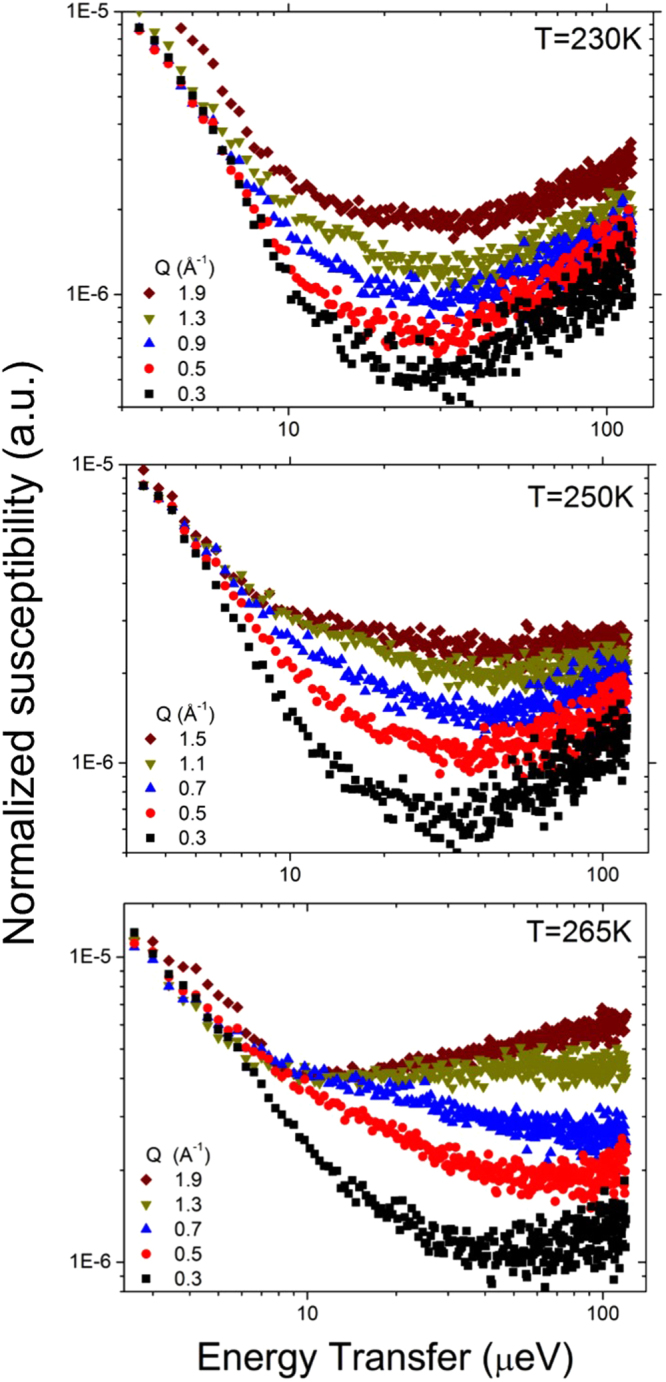
Susceptibility representation of the QENS spectra of hydrated cellulose at 230, 250 and 265 K for selected Q values.

A trend in the temperature-dependent broadening of the QENS spectra is clearly observable, arising from the increased dynamics of water at higher temperatures. Susceptibility analysis has previously been used to study the hydration water associated with different biomolecular systems^59,68–70^. The susceptibility function (Equation 6) was obtained from the normalized QENS spectra and the data are presented as the imaginary part of the susceptibility (χ”(Q,E) versus E (μeV)) in Fig. 4. The susceptibility for the hydrated cellulose at 230 K is largely independent of Q (except for an intensity scaling factor), suggesting the presence of spatially localized relaxations in the hydration water at this temperature. When the temperature is increased to 250 K, a minimum in the curves at ~35 μeV becomes more prominent with increasing Q (Fig. 4). Besides, the low-energy part of the susceptibility spectra becomes Q-dependent. This indicates a dynamic process at low energy, which enters the experimental window from the low-energy side and evidently shifts to higher energy with Q. We can relate this new low-energy dynamical process to the translational diffusive motion of water as the system heats up. Diffusion of surface water at 240–250 K has previously been demonstrated for sufficiently highly hydrated materials^71^. At 265 K, the susceptibility curves show a minimum that shifts strongly to higher frequency with increasing Q, clearly indicative of translational diffusive motions (Fig. 4). The minimum in the susceptibility spectra indicates well-separated processes related to the diffusion of water associated with cellulose, suggesting that a QENS data fit with two Lorentzian components should be chosen over a fit with a single “stretched” component. Although a fit with two Lorentzian components can be considered a simplification, it is preferred over a single “stretched” component fit when the data are known to exhibit more than one dynamic component^72^.

**Figure 5:**
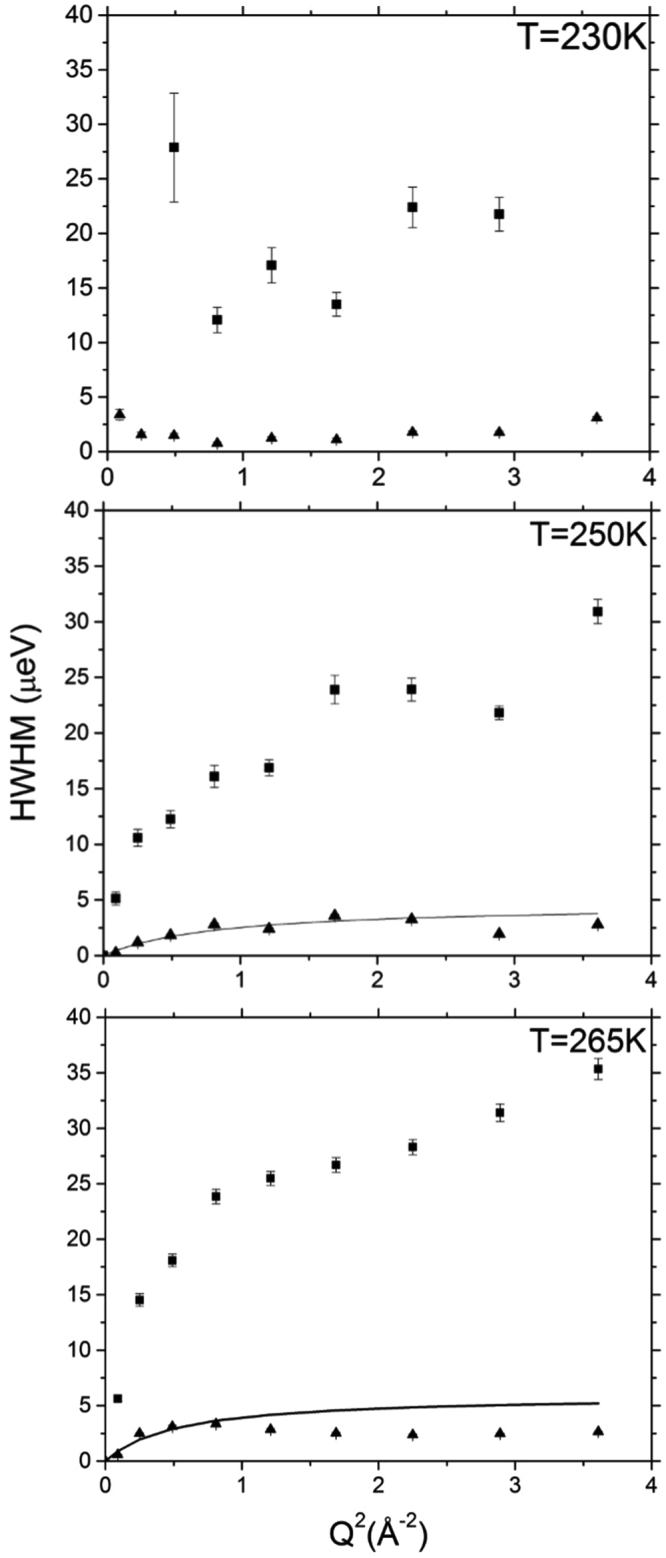
Determination of the diffusion coefficient of bound water. Q^2^ dependence of the broad and narrow component HWHMs from the Lorentzian model at 230, 250 and 265 K are represented by squares and triangles, respectively. The values fitted with the Jump Diffusion Model are drawn as solid lines. The data were fitted up Q = 0.9 Å^−1^, above which there is a significant contribution from the coherent scattering of deuterated cellulose (see text).

The Q-dependence of the Lorentzian HWHMs obtained from the fit to the data measured at 230 K is shown in the upper panel of Fig. 5. As one can expect from the susceptibility spectra, the quasielastic broadening parameters show little Q dependence except for random scatter, indicative of localized motion of water molecules. The data collected at higher temperatures show more systematic Q-dependence. In the two-Lorentzian model fit to the 250 K data set, there is a broader Q-dependent component, which originates from a spatially localized dynamic process (see Supplemental Information for discussion) and a second narrower component approaching zero at low Q that can be attributed to long-range translational diffusion (middle panel of Fig. 5). Similar to the 250 K data set, at 265 K there is a broader component obtained with the two-Lorentzian model that originates from a spatially localized dynamic process (see Supplemental Information for discussion) and the second narrower component that originates from long-range translational diffusion. The suppression of the HWHM at Q values greater than 1.1 Å^−1^, that is especially evident in the narrow Lorentzian component curve is due to a significant contribution from the coherent scattering of the crystalline cellulose (see Fig. 1 panel C).

The Q-dependent narrow components from the Lorentzian model fits (measured at 250 K and 265 K) were quantitatively analyzed to investigate the diffusion of water in cellulose. The Q^2^ – HWHM data for the narrow component were fit by using the Jump Diffusion model^45^ and the fitted parameters are summarized in Table 1. The data were fit only up to Q = 0.9 Å^−1^ due to the interference from the coherent scattering of the crystalline cellulose, as described above. As shown in Table 1, the diffusion coefficients for water at 250 and 260 K are 0.85 ± 0.04 × 10^−10^ m^2^sec^−1^ and 1.77 ± 0.09 × 10^−10^ m^2^sec^−1^, respectively. The previously reported value for the self-diffusion coefficient of supercooled water obtained using pulsed field gradient (PFG) spin-echo (SE) NMR at 268 K is 9.41 × 10^−10^ m^2^sec^−1^ ^73^, providing evidence that the bound water is significantly restricted compared to the bulk. Although there are no other reported values for the diffusion coefficient of water associated with crystalline cellulose, the diffusion coefficient of water associated with saturated cotton linters, obtained using PFG NMR at 291 K, was found to be 6.7 ± 2.2 × 10^−11^ m^2^sec^−1^ ^74^, which is in a similar range to the present values.

**Table 1:** Fit parameters obtained for the narrow component of the two-Lorentzian model using the Jump Diffusion Model.

Temperature (K)	D (10^−10^m^2^sec^−1^)	τ_o_ (ps)	*L* (nm)
250	0.86 ± 0.04	142.2 ± 9.6	0.27
265	1.77 ± 0.09	110 ± 6.6	0.34
268^*^	9.41		

^*^Data from reference^73^.

It should be noted that the diffusion coefficient obtained at 250 K is related exclusively to the translational mobility of “non-freezing bound” water associated with the cellulose surfaces, while the weakly bound water in a confined state trapped between microfibrils remains frozen. The narrow signal component from the two-Lorentzian fit is due to the translational diffusivity of the non-freezing water, while the broader signal component is from the localized in-cage motions (often imprecisely referred to as “rotational” motions) of the same non-freezing water molecules. The diffusion coefficient obtained at 265 K is dominated by the translational mobility of the melted “freezing bound” water trapped between microfibrils. In this case, the narrow and broad signal components are dominated by the translationally diffusing water and the caged water, respectively, of the now highly mobile freezing bound water. Our interpretation of the QENS data is further supported by analysis of the p parameter obtained from the two-Lorentzian Fit (Equation 5), as discussed in the Supplementary Information.

**Figure 6:**
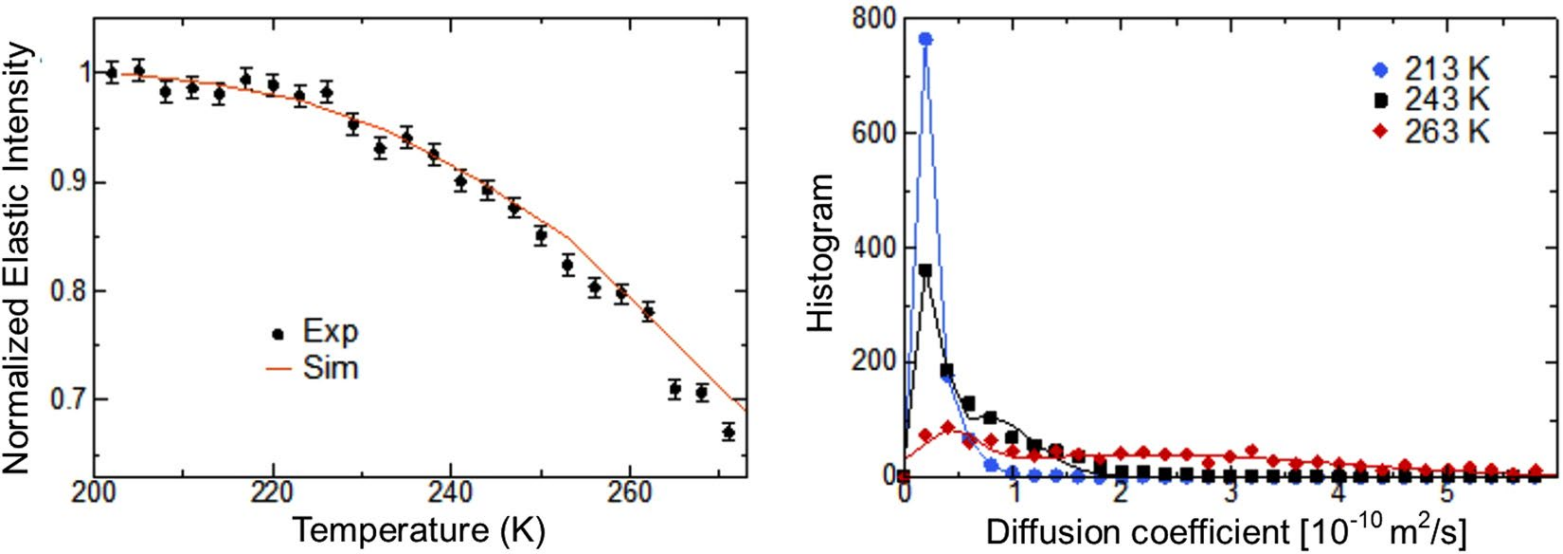
Dynamics of surface water investigated by simulation. *Left* Temperature dependence of the experimental (obtained by fitting lnS_el_ vs Q^2^ over Q = 0.5–0.9 Å^−1^) and simulation-derived mean square displacements. *Right* Probability distribution of 1000 randomly-selected water molecules at three temperatures (symbols) and fits using two Gaussian functions (solid lines).

MD simulations of cellulose fibrils separated by hydration layers were performed to further analyze the properties of the bound water (Fig. 6). The mean square displacement (MSD) of a water molecule, a quantitative measure of its mobility, is calculated as $\langle {\rm{\Delta }}{r}^{2}\rangle =\langle {[r(t)-r(0)]}^{2}\rangle$, where r(t) is the position of the center of mass of the molecule at time t = 800 ps, the FWHM of the resolution of the BASIS spectrometer. To compare the simulations to the experiments, the MSD was calculated from the elastic intensity employing the so-called Gaussian approximation^75^; ${S}_{el}(T)\propto \exp (-{Q}^{2}\langle {\rm{\Delta }}{r}^{2}\rangle /3)$. Good agreement is found between the calculated and measured MSD (Fig. 6). The diffusion coefficient, *D*, is obtained by a linear fit of the MSD vs time: $< {\rm{\Delta }}{r}^{2} > =6Dt$, see Figure S4 in the supplementary Information. The probability distribution (histogram) of the diffusion coefficient of the water molecules was calculated at three temperatures spanning the transitions found in the experiments: T = 213 K, 243 K and 263 K. At all temperatures, the distribution of diffusion coefficients is bimodal and can be fit by a sum of two Gaussian functions to model slow and fast populations of water molecules. The first Gaussian is narrow and its peak position does not vary significantly with temperature. However, its height exhibits a strong temperature dependence, with the population of slow water molecules decreasing from ~85% at 213 K to ~18% at 263 K (Table 2). In contrast, the second Gaussian is much broader. Both the position and the width of the peak increase substantially with temperature, from ~0.5 10^−10^ m^2^/s at 213 K to ~2.2 10^−10^ m^2^/s at 263 K. We suggest this second Gaussian is the freezing population that exhibits long-range translational diffusion while the first Gaussian corresponds to the non-freezing population. Two populations of water molecules are also obtained by examining the probability distribution of the atomic MSD (see Figure S5), in qualitative agreement with Fig. 6. In summary, the results of the simulation are in excellent agreement with the measured elastic intensities and support that bound water exists in two dynamical states or populations.

**Table 2:** Fit parameters of probability distribution of Fig. 6.

Temperature (K)	w_1_	m_1_ (10^−10^m^2^sec^−1^)	σ_1_ (10^−10^m^2^sec^−1^)	w_2_	m_2_ (10^−10^m^2^sec^−1^)	σ_2_ (10^−10^m^2^sec^−1^)
213	0.85	0.25	0.07	0.15	0.52	0.18
243	0.45	0.27	0.07	0.55	0.74	0.43
263	0.18	0.44	0.24	0.82	2.21	1.76

w is the weight, m the mean and σ the standard deviation of the first and second Gaussian distributions.

The QENS and MD simulation results provide clarity to previously reported studies investigating the state of bound water associated with cellulose. In particular, DSC has been widely used to study water associated with the plant cell wall and isolated cell wall component polymers. However, there is controversy about the existence of more than one bound water state associated with cellulose based on DSC data. Nakamura and coworkers^13^, proposed two populations of bound water, that they categorized as freezing and non-freezing components based on analysis of a variety of cellulosic materials at different hydration levels. Accordingly, they assigned the “freezing bound” component as a relatively weakly-bound water population present as clusters or associated with voids and the “non-freezing bound” component as water that is directly associated with the surface of cellulose. The freezing bound water exhibits a phase change between 263–253 K and non-freezing bound water does not freeze down to 203 K^13^. Other studies identified similar behavior in chemically modified cellulose^76,77^ and in intact wood samples^78–80^. However, a more recent work of loblolly pine and isolated cell wall components from the same parent material disputes the existence of two populations of bound water and proposed the detection of the freezing bound water component may depend on the sample preparation method and moisture content.^33^. The authors suggest that the detection of a freezing bound water component is likely due to the homogeneous nucleation of bulk water in the sample and propose that only one type of bound water, the non-freezing component, is present in wood^33^.

Nevertheless, the data presented in the present work exhibit transition temperatures for bound water at ~220 and 260 K, values similar to those found by Nakamura and coworkers^13^. Furthermore, two-dimensional *T*1-*T*2 1 H NMR of spruce wood hydrated under controlled conditions reveals that bound water divides into two components with comparable *T*2, but different *T*1 relaxation times. The NMR data were interpreted as two sub-populations of bound water components that have relaxation parameters consistent with mobile water, such as water in small voids, and a second component that is more consistent with less mobile water that swells wood polymers^17^. In addition,^1^H magic angle spinning NMR experiments of hydrated cotton provided evidence of a range of correlations for bound water, indicative of molecular motions ranging from restricted to relatively mobile^81^. Together, these studies present a strong argument of more than one type of bound water in the plant cell wall. This is reasonable when we consider that cellulose has amorphous and crystalline regions in individual microfibrils that further assemble to form macrofibrils. The different interfaces that this complex supramolecular architecture presents can be expected to influence the binding properties of water to cellulose due to heterogeneity in the local environment. The discrepancies observed between different DSC studies may be a result of experimental parameters such as initial hydration level and heating and cooling rates that can profoundly affect the results obtained, as discussed in a recent review article on wood-water relations^34^.

The microscopic details of water – cellulose interactions give insight into water’s role in biomass pretreatment processes. A recent study demonstrated that a significantly higher glucose yield can be obtained from steam-explosion pretreated biomass when the initial hydration level is below the FSP compared to glucose yields obtained at hydration levels above the FSP^82^. By correlating dynamic water states to biomass mechanical properties, the authors inferred that below the FSP bound water acts as a plasticizer that reduced the mechanical strength of fibers during pretreatment while, at higher hydration levels this effect is mitigated by the presence of free water. However, based on the data presented here, we can propose that the rapid heating of water confined in nanopores in the cellulose below the FSP might also contribute to the disruption of cellulose organization and reduction in the mechanical strength of the fibers. Other work that described the molecular level details of the structural changes in biomass during pretreatment using multiple structural techniques combined with MD simulations showed the coalescence of cellulose microfibrils occurred due to expulsion of water from the interfibrillar space, leading to the hypothesis of a balance between the entropy and enthalpy of hydration as a unifying principle for overcoming kinetic barriers during pretreatment^7^. These studies highlight the critical role that water plays both at the microscopic and macroscopic scales in biomass deconstruction during thermochemical pretreatment processes.

## Conclusions

The relationship between cellulose supramolecular structure and the types of interfaces it forms with water is important in many applications. Understanding how water is distributed in the cell wall is critical for developing accurate models of its hydration water that can inform on fundamental questions about cellulose - water interactions. Additionally, the reactivity of cellulose is intimately connected to its interfacial properties, and understanding water’s role in this environment will help to discover the underlying processes that change biomass morphology during different pretreatment regimes for biofuels production. The present study presents the first systematic investigation of cellulose – water interactions using QENS and MD simulation.

An overall picture of the temperature dependent processes that occur in hydrated cellulose emerges as follows. The water trapped between microfibrils melts or freezes at ca. ~260 K. At lower temperatures, the remaining surface water does not freeze, but instead gradually becomes a supercooled liquid, and shows progressively slowing dynamics, which below ~220 K could no longer be observed with the resolution of our experiment. Conversely, on warming up from cryogenic (~4 K) temperatures, the dynamics of surface water becomes measurable with the resolution of the BASIS above ~220 K. As the interfibrillar water is still frozen, thus yielding only an elastic scattering signal, the 230 K data set probes the dynamics of the surface water, which at this temperature is already locally mobile and gives rise to some QENS broadening, but this hydration water is not yet long-range translationally mobile in the experimental time window (~100 ps). The presence of translationally mobile water is seen in the 250 K data set, while the interfibrillar water is still frozen. Finally, on further warming up, the interfibrillar water melts, affecting and likely even dominating the 265 K spectra with its prominent long-range translational mobility. Further support for the presence of two dynamic states comes from MD simulations modeling interfibrillar cellulose water. The accuracy of the simulations is verified by the calculation of elastic intensities that are found to be in excellent agreement with experiments. The distribution of diffusion coefficients of water molecules in the simulations is bimodal, implying the existence of two populations of water molecules in the system.

Additional studies are required to determine if the states of water observed in this study for hydrated crystalline cellulose can also be discerned in intact wood samples using QENS. However, based on our data and recent 2D NMR experiments^17^ it is reasonable to suggest that the description of only two kinds of water present in native wood, namely free water in the cell lumens and non-freezing bound water inside the cell walls^34^, may not accurately describe how water interacts with the plant cell wall.

## Electronic supplementary material

supplementary information
