# Supplementary material for: Dynamics of water bound to crystalline cellulose
Source: Sci Rep. 2017 Sep 19;7:11840. doi: 10.1038/s41598-017-12035-w (PMC5605533; doi:10.1038/s41598-017-12035-w)
Supplement: Supplementary file 1 — supplementary information [file 41598_2017_12035_MOESM1_ESM.pdf]

SUPPLEMENTARY INFORMATION

Dynamics of water bound to crystalline cellulose

Hugh O'Neill[1,5*], Sai Venkatesh Pingali[1], Loukas Petridis[2], Junhong He[1], Eugene Mamontov[3], Liang Hong[6], Volker Urban[1], Barbara Evans[4], Paul Langan[1], Jeremy C. Smith[2,5], and Brian H. Davison[2]

[1]Biology and Soft Matter Division, [2]Biosciences Division, [3]Chemical and Engineering Materials Division, [4]Chemical Sciences Division, Oak Ridge National Laboratory, Oak Ridge, Tennessee 37831, United States

[5]Department of Biochemistry and Cellular and Molecular Biology, University of Tennessee, Knoxville, Tennessee 37996, United States

[6] Department of Physics and Astronomy & Institute of Natural Sciences, Shanghai Jiao Tong University, Shanghai 200240, China

Elastic Intensity Structure Factor (EISF)

The EISF (Q) plot for the 230K, 250K, and 265K are presented in Figure S1. Unfortunately, the EISF plots provide rather limited information in this study, beyond that the signal becomes progressively less elastic as the temperature is raised. This is because the cellulose itself makes a large contribution to the elastic scattering, thereby making the EISF(Q) plots rather flat. In addition, the EISF(Q) would provide valuable information for truly confined water, which we believe was not the case, especially for the 250 K and 265 K measurements because the water measured at those temperatures was translationally mobile.

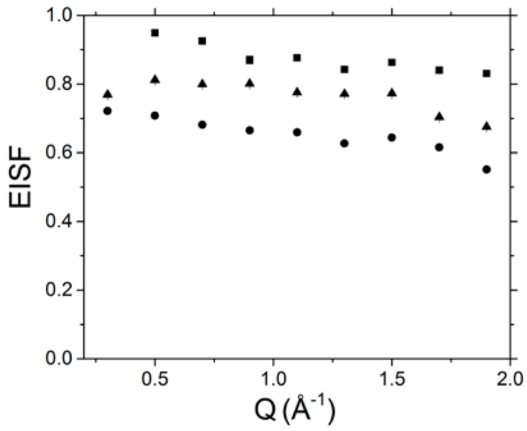

Figure S1: Q dependence of the EISF parameter from the two-Lorentzian fit model at 230, 250 and 265 K are represented by squares, triangles, and circles, respectively.

Analysis of the broad component of the two-Lorentzian model

The analysis of HWHM of the broad Lorentzian component is presented below. Although, it is possible to obtain a reasonable fit to these data using the Jump Diffusion model the physical meaning of the values obtained is not related to the true diffusivities. We interpret the broad Lorentzian component as the contribution from the localized or caged dynamics of water (often referred to as "rotational water diffusivity" in the older literature) associated with cellulose. The fact that the values obtained for the broad component at 250 and 265 K are similar is consistent with the notion that rotational or caged dynamics shows a weak temperature dependence, as has previously been described for the translational dynamics of the hydration water of proteins (Tournier, A.L., Xu, J. and Smith, J.C. "Translational Hydration Water Dynamics Drives the Protein Glass Transition" *Biophysical Journal* **85** 1871–1875 (2003)).

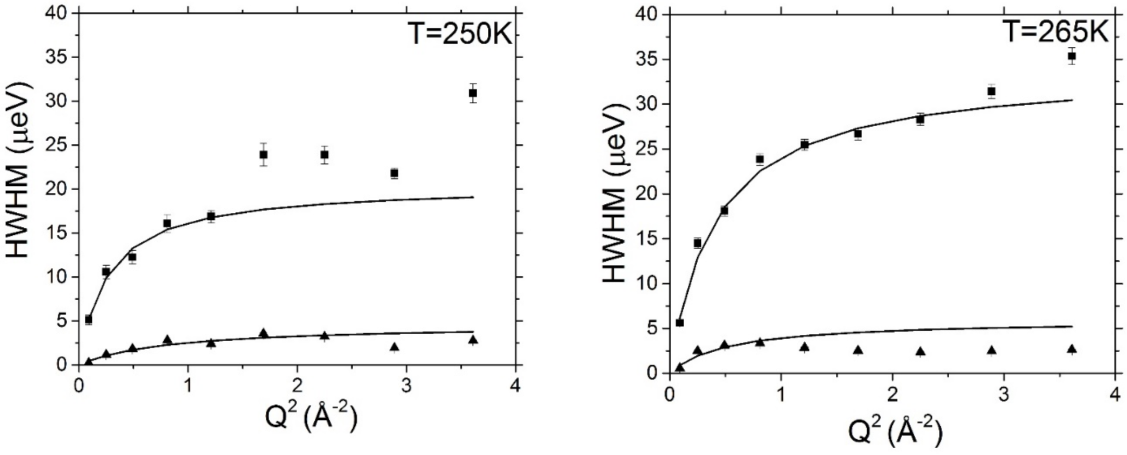

Figure S2. Determination of the diffusion coefficient of bound water**.** Q^2 dependence of the broad and narrow component HWHMs from the Lorentzian model at 250 and 265 K are represented by squares and triangles, respectively. The values fitted with the Jump Diffusion Model are drawn as solid lines. The data were fitted up Q = 0.9 Å^{-1}, above which there is a significant contribution from the coherent scattering of deuterated cellulose (see text).

Table S1: Fit parameters obtained for broad components of the two-Lorentzian model using the Jump Diffusion Model

Temperature (K)	D (10^{-10}m^{2}sec^{-1})	τ_o (ps)	*L* (nm)
250	11.7 ± 1.45	32.2 ± 1.9	0.48
265	12.7 ± 0.52	19.4 ± 0.42	0.38

Analysis of P(Q) parameter from the two-Lorentzian model

Our interpretation of the QENS data is further supported by analysis of the p parameter obtained from the two-Lorentzian fit (see Equation 5, main text). This parameter is interpreted as EISF for the transient confinement volume of a water molecule through the caging by the neighboring water molecules. The Q-dependence of p for values < 1 Å^{-1} (due to interference from the crystalline cellulose) is an indication that the two Lorentzian components of the signal are not related to two separate water populations, but only to one water population at a given temperature. At 250 K, it is exclusively the non-freezing water. At 265 K, it is dominated by the highly mobile freezing water. The 230 K data are too dynamically suppressed to draw a decisive conclusion, but they are qualitatively similar to the 250 K data, with only the non-freezing water being dynamically active. If the narrow and broad components obtained at any given temperature were due to two separate populations of water then the p parameter would be Q-independent, as is discussed elsewhere [Qvist, J., Schober, H. & Halle, B. Structural Dynamics of Supercooled Water from Quasielastic Neutron Scattering and Molecular Simulations. *J. Chem. Phys*. **134**, 144508 (2011)].

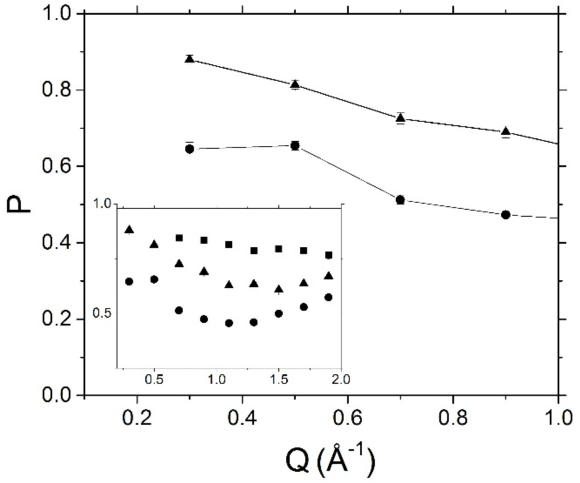

Figure S3. Q dependence of the P parameter from the Lorentzian model at 250 and 265 K are represented by triangles, and circles, respectively. *Inset*: Same data with Q range extended to 1.9 Å^{-1}. The data at 230, 250, and 265K are represented by squares, triangles, and circles, respectively.

Calculation of diffusion coefficient from calculated Mean Square Displacements

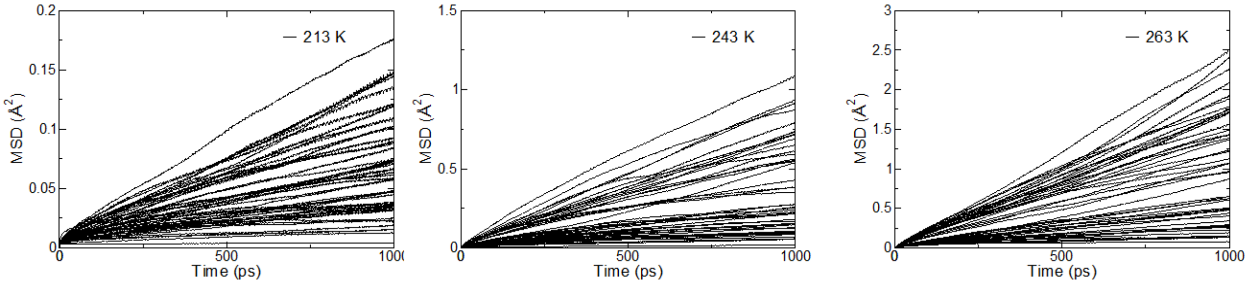

Figure S4: Mean square displacement as a function of time for 50 randomly-chosen water molecules at 213 K (left), 243 K (middle) and 263 K (right).

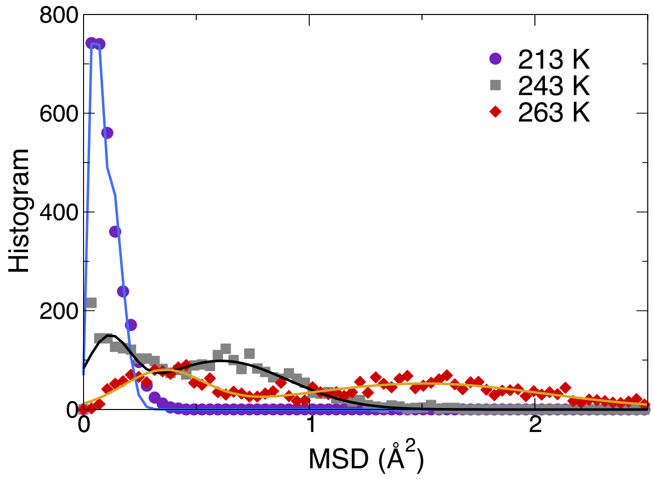

Figure S5: Histogram of the mean square displacement (at 800 ps) for 3000 randomly-chosen water molecules (symbols) as well as a two Gaussian fit (lines). Each Gaussian has weight w, mean m and width σ.

Temperature (K)	w_1	m_1 (Å^2)	σ_1 (Å^2)	w_2	m_2 (Å^2)	σ_2 (Å^2)
213	0.40	0.05	0.013	0.60	0.52	0.11
243	0.30	0.11	0.11	0.70	0.74	0.70
263	0.32	0.35	0.17	0.68	2.21	1.51
